# Complex Evaluation of Tissue Factors in Pediatric Cholesteatoma

**DOI:** 10.3390/children8100926

**Published:** 2021-10-16

**Authors:** Kristaps Dambergs, Gunta Sumeraga, Māra Pilmane

**Affiliations:** 1 Department of Otorhinolaryngology, Riga Stradiņš University, LV-1002 Riga, Latvia; gunta.sumeraga@rsu.lv; 2 Department of Morphology, Institute of Anatomy and Anthropology, Riga Stradiņš University, LV-1007 Riga, Latvia; mara.pilmane@rsu.lv

**Keywords:** cholesteatoma, metalloproteases, sonic hedgehog, cytokines, transcription factors, Ki-67, vascular endothelial growth factor, defensins, children

## Abstract

The aim of this study was to describe the appearance and distribution of tissue remodeling markers (MMP-2, MMP-9, TIMP-2, TIMP-4), Sonic hedgehog gene protein (Shh), pro- and anti-inflammatory cytokines (IL–1, IL–10), transcription factor (NF-κβ), proliferation marker (Ki–67), angiogenetic factor (VEGF), tissue defensins (HβD–2, HβD–4) of the pediatric cholesteatoma. Sixteen cholesteatoma samples were obtained from children, eleven skin controls from cadavers. Tissues were stained for MMP-2, MMP-9, TIMP-2, TIMP-4, Shh, IL–1, IL–10, NF-κβ, Ki–67, VEGF, HβD–2, HβD–4. Non-parametric statistic, Mann–Whitney, and Spearman’s coefficient was used. A statistically significant difference was seen between Shh and HβD–2 in perimatrix and control connective tissue, between NF-κβ in cholesteatoma and control skin, and between HβD–4 in matrix and skin epithelium. Complex intercorrelations between MMPs, NF-κβ and VEGF cause the intensification of angiogenesis in cholesteatoma. The persistent increase in Shh gene protein expression in cholesteatoma perimatrix suggests the stimulation of the cholesteatoma growth in children. Similar expression of IL-1 and IL-10 and their intercorrelation, proves there is a balance between pro- and anti-inflammatory cytokines. NF-κβ, and not Ki-67, seems to be the main inducer of cellular proliferation. The main antimicrobial protection is provided by HβD-2.

## 1. Introduction

Cholesteatoma is a locally destructive and hyperproliferative but benign lesion composed of a stratified keratinizing squamous epithelium found mostly in the middle ear [1]. Cholesteatoma is considered to be a rare lesion. In the early 2000s, the incidence of pediatric cholesteatoma was 3 per 100,000 [2]. This rarity is one reason why there are many uncertainties about the development and pathological growth of cholesteatoma in the middle ear.

Two of the enzymes proven to play an important role in the pathogenesis of cholesteatoma are Matrix metalloproteinase-2 (MMP-2) and Matrix metalloproteinase-9 (MMP-9). They are responsible for the degradation of the ECM (extracellular matrix) [3]. MMP-2 and MMP-9 are in the same group of MMPs (gelatinases) and can cleave collagen [4].

In cholesteatoma, MMP-2 plays a major role in bone resorption and angiogenesis, which is one of the main factors under study when researchers analyze the aggressiveness of cholesteatoma [5,6]. MMP-9 is strongly associated with angiogenesis and is specifically seen in areas with inflammatory cell infiltration [7,8]. Normal tissue remodulation is achieved by a balance between MMPs and tissue inhibitors of metalloproteinases (TIMPs). It is known and proven that an imbalance between MMPs and TIMPs triggers destructive processes in cholesteatoma patients [9]. TIMP-2 is expressed by osteoclasts and osteoblasts, and these cells secrete MMP-2 and MMP-9 [9]. TIMPs act as specific inhibitors of MMPs’ enzymatic activity, and if there is an imbalance, then bone remodulation is activated in middle ear structures. Yet, it is still unclear how exactly TIMP-2 affects MMP-2 or MMP-9 in cholesteatoma tissue [10]. As well as TIMP-2, TIMP-4 also acts as an inhibitor of MMP-2 and MMP-9 in different tissues and suppresses the growth of various tumors [11]. Still, there are no data available on how TIMP-4 acts in cholesteatoma tissue.

The Sonic hedgehog (Shh) gene protein might be involved in the genetic morphopathogenesis of cholesteatoma. The Shh gene in the human body is responsible for the development of the first pharyngeal arch, from which the external ear canal evolves [12,13,14,15]. This is critical because the external ear canal originates from where the skin epithelium migrates in the middle ear and forms an acquired cholesteatoma [16]. There is no information available in scientific databases on whether Shh is important in the ontogenesis of cholesteatoma.

A persistent cytokine response has always been mentioned in cholesteatoma in relation to the inflammatory process in the matrix and perimatrix. Pathological hyperproliferation of keratinocytes through a complex cascade induces the release of pro-inflammatory cytokine Interleukin-1 (IL-1) in the cholesteatoma matrix [17,18]. IL-1—acting through fibroblasts, osteoclasts, osteoblasts, macrophages and prostaglandins—causes degradation of the bone matrix [19]. The main anti-inflammatory cytokine, Interleukin-10 (IL-10), on the other hand, works against high pro-inflammatory cytokine levels in cholesteatoma tissue [17,18]. An imbalance of IL-1 and IL-10 is believed to cause an uncontrolled inflammatory process, which is destructive for the surrounding bone in the middle ear [20].

The most obvious abnormality in cholesteatoma is hyperproliferative epithelial cells in its matrix. Nuclear factor-kappa beta (NF-κβ) is one of the most important factors in cell proliferation, differentiation, inflammation, the immune response, carcinogenesis and protection against apoptosis, and is one of the components responsible for the development of cholesteatoma [21]. Li et al. showed that NF-κβ is upregulated in cholesteatoma tissue compared to the unchanged skin epithelium [22]. Additionally, Ki-67 is located in the cell nucleus in all proliferative cells. It is present in all phases of the cell cycle except for G0 [23], and has proven to be useful for indicating cell proliferation in cholesteatoma [24]. Hamajima et al. showed that NF-κβ and Ki-67 act together in one pathway to increase cell proliferation and aggressiveness in cholesteatoma [25].

Furthermore, neo-angiogenesis in the perimatrix is an important factor for the continuous growth of cholesteatoma [26] and, therefore, is responsible for the aggressiveness toward the surrounding tissue in the temporal bone [27]. Vascular endothelial growth factor (VEGF) is believed to be one of the most potent angiogenetic factors in chronic ear infections with cholesteatoma [28]. Even though VEGF was first found in endothelial cells, it was later proven that it is also detected, for example, in keratinocytes [29]. Further to this, Fukodome et al. hypothesized that VEGF could also be secreted by keratocytes from the cholesteatoma matrix and released in the perimatrix to induce angiogenesis in a paracrine manner [27]. However, uncertainties remain about how VEGF functions in this pathological process.

Human beta defensin-2 and -4 are known for their antibacterial properties in human tissue. Clinically, chronic middle ear infection with cholesteatoma is often seen to become inflamed. It is assumed that infection could accelerate its growth and reoccurrence [30,31]. These bacterial infections, in the majority of cases, are caused by *P. aeruginosa* [32], which is proven to be a powerful inducer of Human beta defensin-2 (HβD-2) and Human beta defensin-4 (HβD-4) [33,34]. Park et al. found that HβD-2 is overexpressed in cholesteatoma tissue compared to unchanged skin epithelium [35]. Additionally, HβD-2 and HβD-4 are known to be secreted by keratinocytes [36]. However, there are limited data on how HβD-2 affects pediatric cholesteatoma, and there are no studies available about the relations between HβD-4 and middle-ear cholesteatoma.

Thus, the aim of this study was to describe the appearance, distribution and possible clinically significant correlations of tissue remodeling markers (MMP-2, MMP-9, TIMP-2, TIMP-4), Sonic hedgehog gene protein (Shh), pro- and anti-inflammatory cytokines (IL–1, IL–10), transcription factor (NF-κβ), proliferation marker (Ki–67), angiogenetic factor (VEGF) and local tissue defensins (HβD–2, HβD–4) of the pediatric cholesteatoma tissue compared to control skin tissue.

## 2. Materials and Methods

### 2.1. Tissue Samples

Cholesteatoma specimens were retrieved during cholesteatoma surgery at the Children’s Clinical University Hospital, Riga, Latvia, but the morphological analysis and immunochemical staining of the tissue were conducted at the Department of Morphology of Riga Stradiņš University, Riga, Latvia. Nineteen cholesteatoma tissue samples were obtained from children during cholesteatoma surgery, from nine males and ten females (aged 6–17 years, mean age 12.56 years). Fourteen deep external meatal skin controls were obtained from fourteen different cadavers in a collection of the Institute of Anatomy and Anthropology; ten were adults (aged 45–70 years), four were children (aged 12–14 years) and no chronic ear diseases were documented.

Three patients were excluded from the study due to incomplete cholesteatoma material, which was invalid for immunohistochemical analysis. Three control group skin samples were further excluded because of insufficient skin material, which was also invalid for immunohistochemical analysis.

This study was approved by the local Ethical Committee of Riga Stradiņš University (05.09.2019; no. 6-2/7/4). All of the patients or their parents gave informed consent to participate in the study. The nature of the study was fully explained to the patients and their parents.

### 2.2. Immunohistochemical Analysis

The tissues were fixed in a mixture of 2% formaldehyde and 0.2% picric acid in 0.1 M phosphate buffer (pH 7.2). Afterward, they were rinsed in Tyrode buffer (content: NaCl, KCl, CaCl2_2H2O, MgCl2_6H2O, NaHCO3, NaH2PO4_H2O, glucose) containing 10% saccharose for 12 h and then embedded in the paraffin.

Thin sections (3 µm) were cut, which were then stained with hematoxylin and eosin for routine morphological evaluation. The Biotin-Streptavidin biochemical method was used for immunohistochemistry (IMH) to detect: Matrix metalloproteinase-2 (MMP-2; cat. no. AF902, LOT DUBO 34081, obtained from goat, 1:100 dilution, R&D Systems, Germany); Matrix metalloproteinase-9 (MMP-9; sc-10737, rabbit, working dilution 1:100, Santa Cruz Biotechnology, Inc., Santa Cruz, CA, USA); Tissue inhibitor of metalloproteinase-2 (TIMP-2; cat. no. 3A4, sc-21735, obtained from mouse, 1:200 dilution, Santa Cruz Biotechnology, Inc. Dallas, TX, USA); Tissue inhibitor of metalloproteinase-4 (TIMP-4; at 1:100 sc-30076, rabbit, working dilution 1:100, Santa Cruz Biotechnology, Inc.); Sonic hedgehog (Shh; mouse; AF 464, working dilution 1:60, R&D Systems, Germany); Interleukine-1 (IL-1; orb308737, working dilution 1:100, Biorbyt Ltd., Cambridge, UK); Interleukine-10 (L-10; 250713, working dilution 1:100, BioSite, Täby, Sweden); Nuclear factor-kappa beta (NFkB-105; obtained from rabbit, 1:100 dilution, Abcam, UK); Ki-67 (1508202A, working dilution 1:100, Sigma-Aldrich, St. Louis, MO, USA); Vascular endothelial growth factor (VEGF; orb191500, rabbit, polyclonal, working dilution 1:100, Biorbyt Ltd.); Human beta defensin-2 (HβD-2; goat; 1:100; Bio-Techne, UK); Human beta defensin-4 (HβD-4; mouse; 1:100; Santa Cruz Biotechnology, Inc. Dallas, TX, USA).

The slides were analyzed via light microscopy by two independent morphologists using a semi-quantitative method [37]. The results were evaluated by grading the appearance of positively stained cells in the visual field. Structures in the visual field were labeled as follows: 0, no positive structures; 0/+, occasional positive structures; +, few positive structures; +/++, few-to-moderate positive structures; ++, moderate positive structures; ++/+++, moderate-to-numerous positive structures; +++, numerous positive structures; +++/++++, numerous-to-abundant structures; ++++, an abundance of positive structures in the visual field.

For visual illustration, a Leica DC 300F digital camera and the image processing and analysis software Image-Pro Plus (Media Cybernetics, Inc., Rockville, MD, USA) were used.

### 2.3. Statistical Analysis

Data processing was performed with SPSS software, version 22.0 (IBM Company, Chicago, IL, USA). Spearman’s rank correlation coefficient was used to determine correlations between factors, where r = 0–0.2 was assumed as a very weak correlation, r = 0.2–0.4 a weak correlation, r = 0.4–0.6 a moderate correlation, r = 0.6–0.8 a strong correlation and r = 0.8–1.0 a very strong correlation. To analyze the control group versus patient data, the Mann–Whitney U test was used. The levels of significance for the tests were chosen as 5% and 1% (*p*-values < 0.05 and <0.01).

## 3. Results

### 3.1. Findings of Routine Histological Analysis

**Figure 1:**
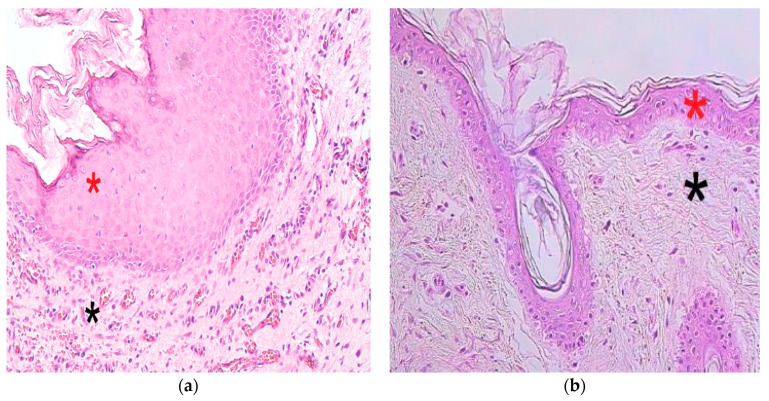
Micrographs of cholesteatoma and control skin tissue. (**a**) The cystic layer of cholesteatoma mostly consists of desquamated, anucleate keratin mass, matrix (*) with hyperproliferative stratified squamous epithelium and perimatrix (*), subepithelial connective tissue, with some consisting of inflammatory cells and blood vessels. Haematoxylin and eosin, X 200; (**b**) Control material demonstrates unchanged skin epithelium (*) and connective tissue (*). Haematoxylin and eosin, X 200 [38].

Cholesteatoma tissue presented anucleate keratin squames, which are a primary component of cholesteatoma and form the cystic layer. The middle part (matrix) consisted of hyperproliferative stratified squamous epithelium, and the outermost part (perimatrix) was inflamed subepithelial connective tissue or granulation tissue composed of inflammatory cells—such as lymphocytes, plasma cells and neutrophil leucocytes—along with collagen fibers, fibrocytes and many small blood vessels (Figure 1a). The control group tissue from the deep external ear canal skin demonstrated an unchanged stratified squamous epithelium and subepithelial connective tissue without inflammation (Figure 1b).

### 3.2. Immunohistochemistry Findings for Tissue Remodeling Factors

**Figure 2:**
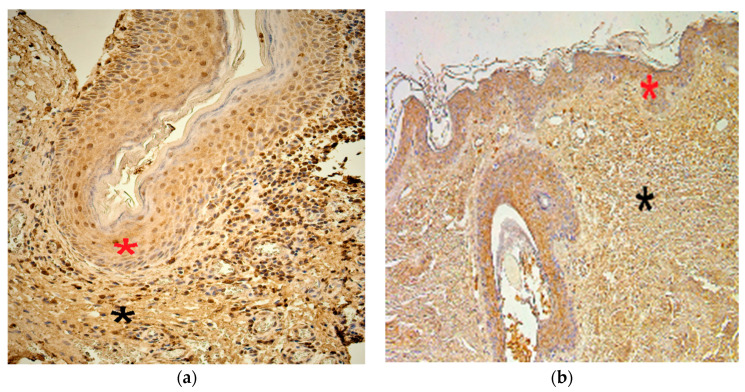
Immunohistochemical micrographs of cholesteatoma tissue and control group. (**a**) Note a few to moderate MMP–2 positive cells in matrix (*) and moderate in the perimatrix (*). MMP–2 IHC, X 200; (**b**) Note a numerous MMP–2 positive cells in the epithelium (*) and a few in the connective tissue (*) of a control skin sample, MMP–2 IHC, X 200 [38].

The numbers of MMP-2-positive cells in the cholesteatoma matrix ranged from a lack of positive cells (0) to moderate or numerous positive cells (++/+++). In the perimatrix, MMP-2-positive cells ranged from none (0) to moderate (++), in the control group, MMP-2-positive cells in the epithelium ranged from none (0) to numerous (+++) and in the connective tissue, there were a few (+) MMP-2-positive cells (Figure 2a,b).

**Figure 3:**
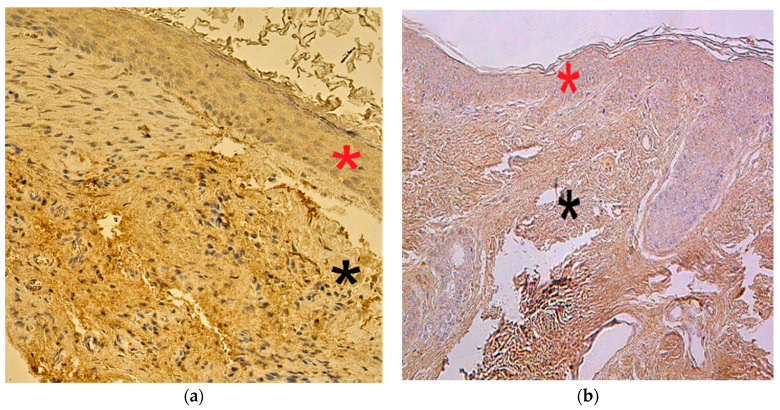
Immunohistochemical micrographs of cholesteatoma tissue and control group. (**a**) Moderate MMP-9 positive cells in matrix (*) and a few to moderate in the perimatrix (*) of a cholesteatoma patient, MMP-9 IHC, X 200; (**b**) Moderate MMP-9 positive cells in the epithelium (*) and a few to moderate in the connective tissue (*) of a control skin sample, MMP-9 IHC, X 200 [38].

The appearance and distribution of MMP-9 immunoreactive cells in the matrix and perimatrix were marked by a range from none (0) to moderate (++), while in the control group, the distribution ranged from occasional (0/+) to moderate (++) positive cells (Figure 3a,b).

**Figure 4:**
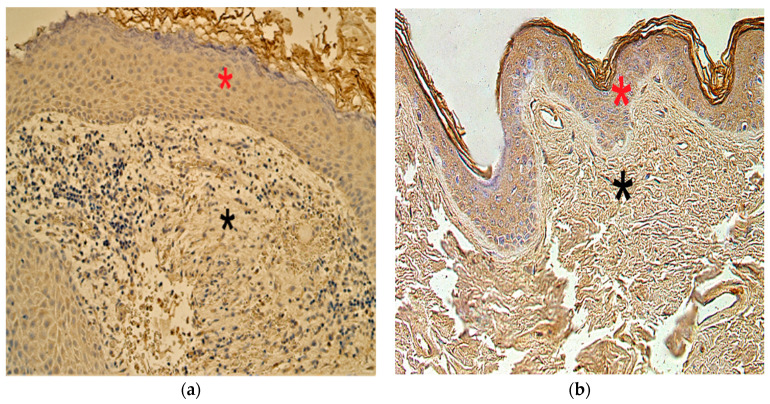
Immunohistochemical micrographs of cholesteatoma tissue and control group. (**a**) Few to moderate TIMP–2 positive cells in the matrix (*) and occasional in the perimatrix (*), TIMP–2 IHC, X 200; (**b**) Moderate to numerous TIMP–2 positive cells in the epithelium (*) and a few in the connective tissue (*) of a control skin sample, TIMP–2 IHC, X 200 [38].

TIMP-2 presented variance in the cholesteatoma matrix and perimatrix, ranging from a lack of positive cells (0) to numerous (+++) immunoreactive cells. In the control tissue, the distribution varied from no TIMP-2-containing cells (0) to moderate-to-numerous (++/+++) positive cells (Figure 4a,b).

**Figure 5:**
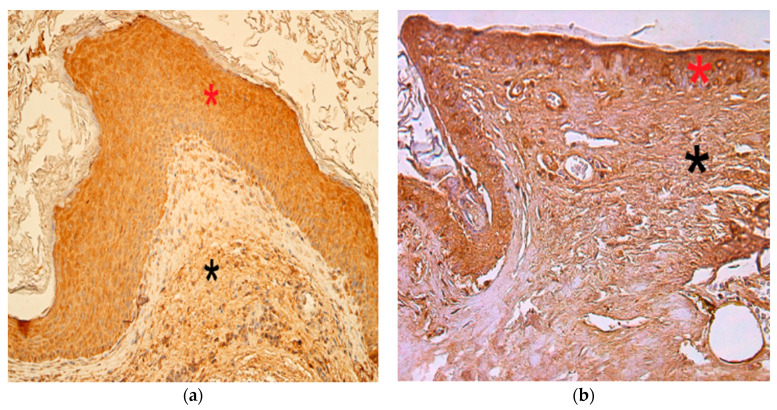
Immunohistochemical micrographs of cholesteatoma tissue and control group subjects. (**a**) Numerous to abundant TIMP-4 positive cells in matrix (*) and numerous in the perimatrix (*) of a cholesteatoma patient, TIMP-4 IHC, X 200; (**b**) Moderate to numerous TIMP-4 positive cells in the epithelium (*) and moderate in the connective tissue (*) of a control skin sample, TIMP-4 IHC, X 200 [38].

The numbers of TIMP-4-containing cells in cholesteatoma varied from occasional (0/+) to numerous-to-abundant (+++/++++). In the control group, the range was from a few (+) to numerous (+++) TIMP-4 immunoreactive cells (Figure 5a,b).

### 3.3. Immunohistochemistry Findings for Shh Gene Protein

**Figure 6:**
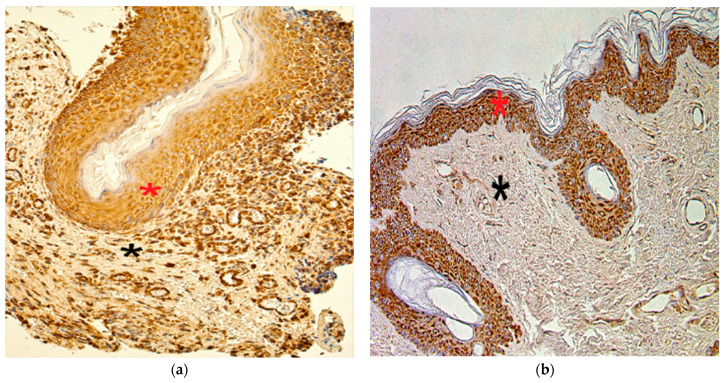
Immunohistochemical micrographs of cholesteatoma tissue and control group subjects. (**a**) Numerous Shh positive cells in matrix (*) and moderate in the perimatrix (*) of a cholesteatoma patient, Shh IHC, X 200; (**b**) Numerous to abundance Shh positive cells in the epithelium (*) and a few in the connective tissue (*) of a control skin sample, Shh IHC, X 200 [38].

Shh gene protein-positive cells in the matrix and perimatrix marked a range from none (0) to numerous (+++). In the control skin epithelium and connective tissue, Shh-reactive cells varied from none (0) to numerous-to-abundant (+++/++++) (Figure 6a,b).

### 3.4. Immunohistochemistry Findings for Pro- and Anti-Inflammatory Cytokines

**Figure 7:**
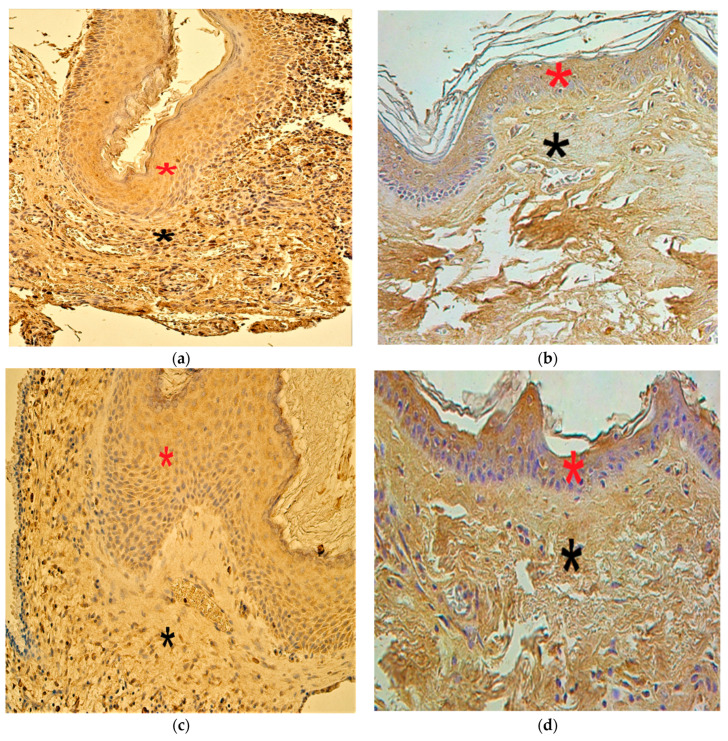
Immunohistochemical micrographs of cholesteatoma tissue and control group. (**a**) Numerous IL–1 positive cells in the matrix (*) and moderate in the perimatrix (*). IL–1 IHC, X 200; (**b**) Moderate IL–1 positive cells in the epithelium (*) and a few in the connective tissue (*) of a control skin sample, IL–1 IHC, X 200; (**c**) Numerous IL–10 positive cells in the matrix (*) and moderate in the perimatrix (*). IL–10 IHC, X 200; (**d**) Moderate IL–10 positive cells in the epithelium (*) and moderate in the connective tissue (*) of a control skin sample, IL–10 IHC, X 200 [38].

In the patient group, the cytokine IL-1 and anti-inflammatory cytokine IL-10 findings demonstrated a range from occasional (0/+) to numerous (+++) positive cells. In the control group, IL-1-containing cells ranged from none (0) to moderate-to-numerous (++/+++) and the IL-10-positive cells from a few (+) to numerous (+++) (Figure 7a–d).

### 3.5. Immunohistochemistry Findings for Cellular Proliferation Markers

**Figure 8:**
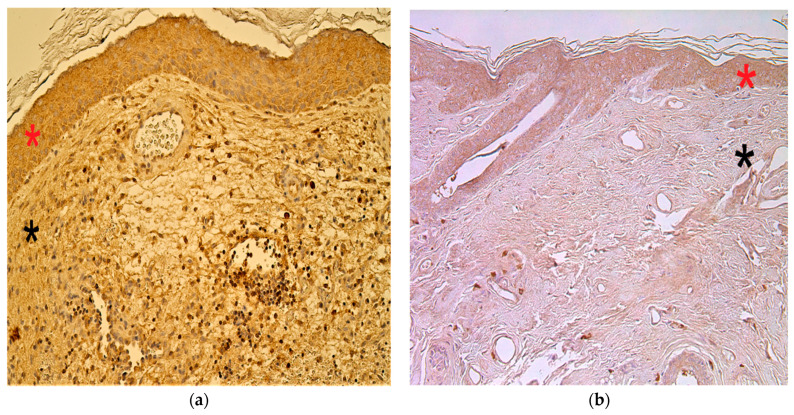
Immunohistochemical micrographs of cholesteatoma tissue and control group. (**a**) Moderate to numerous NF-κβ positive cells in matrix (*) and moderate in the perimatrix (*) of a cholesteatoma patient, NF-κβ IHC, X 200; (**b**) Moderate NF-κβ positive cells in the epithelium (*) and a few to moderate in the connective tissue (*) of a control skin sample, NF-κβ IHC, X 200 [38].

The appearance and distribution of NF-κβ-containing cells in the patient group marked a range from none (0) to numerous (+++). In the control group, NF-κβ positive cells varied from none (0) to moderate (++) (Figure 8a,b).

**Figure 9:**
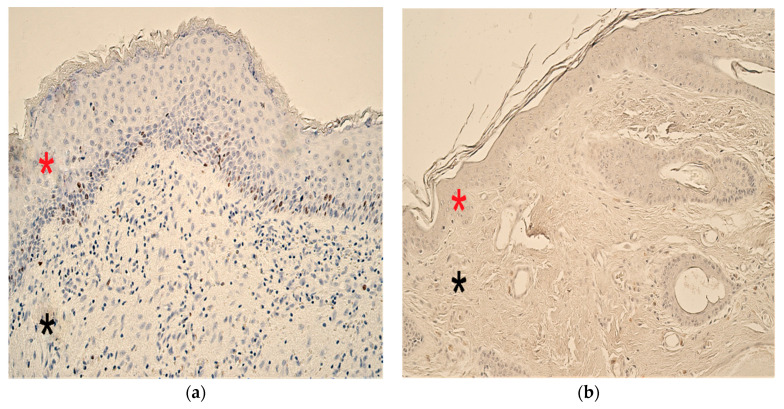
Immunohistochemical micrographs of cholesteatoma tissue and control group subjects. (**a**) A Few (+) Ki-67 positive cells in matrix (*) and occasional in the perimatrix (*) of a cholesteatoma patient, Ki-67 IHC, X 200; (**b**) An occasional Ki-67 positive cells in the epithelium (*) and the connective tissue (*) of a control skin sample, Ki-67 IHC, X 200 [38].

The proliferation marker Ki-67 in cholesteatoma presented variance from no (0) positive cells to a few (+). In the controls, Ki-67-immunoreactive cells ranged from none (0) to occasional (0/+) (Figure 9a,b).

### 3.6. Immunohistochemistry Findings for Angiogenetic Factor

**Figure 10:**
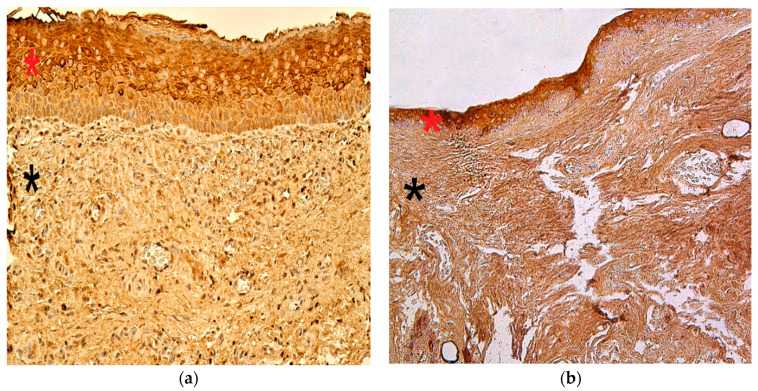
Immunohistochemical micrographs of cholesteatoma tissue and control group subjects. (**a**) Numerous to abundant VEGF positive cells in the matrix (*) and moderate to numerous VEGF positive endothelial cells in the perimatrix (*) of a cholesteatoma patient, VEGF IHC, X 200; (**b**) Moderate to numerous VEGF positive cells in the epithelium and a few VEGF positive endothelium (*) cells of connective tissue (*) of the control skin, VEGF IHC, X 200 [38].

VEGF-positive cells in the matrix and perimatrix were graded with values from none (0) to numerous-to-abundant (+++/++++) positive cells. In the skin epithelium and connective tissue, VEGF immunoreactive cells varied from none (0) to numerous (+++) (Figure 10a,b).

### 3.7. Immunohistochemistry Findings for Human Beta Defensins

**Figure 11:**
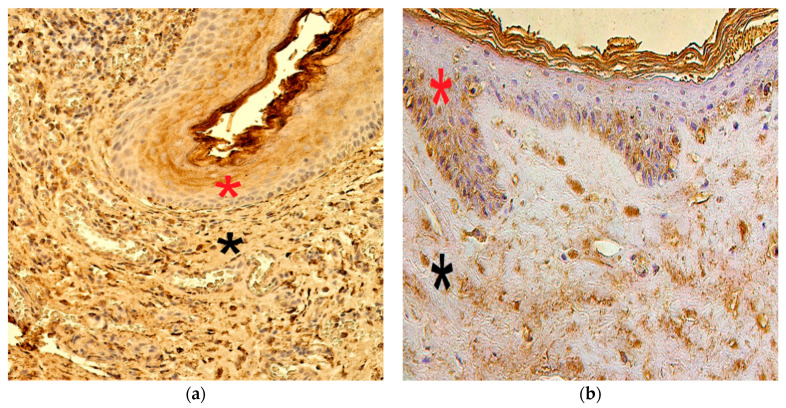

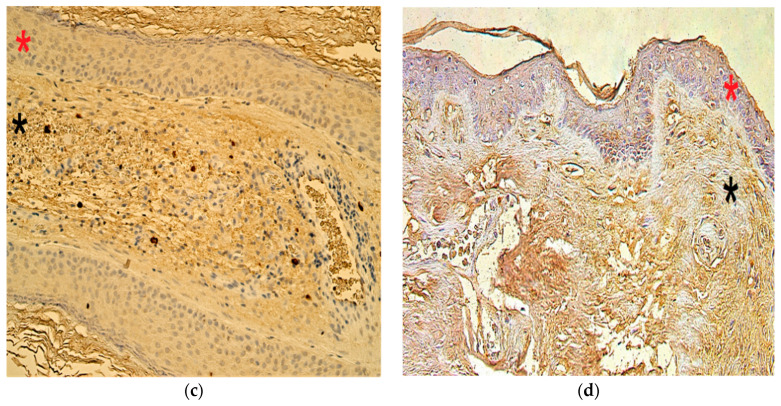
Immunohistochemical micrographs of cholesteatoma tissue. (**a**) Moderate HβD–2 positive cells in a matrix (*) and perimatrix (*) of a cholesteatoma patient. HβD–2 IHC, X 200; (**b**) Few HβD–2 positive cells in the epithelium (*) and occasional in the connective tissue (*) of a control skin sample, HβD–2 IHC, X 200. (**c**) Few to moderate HβD–4 positive cells in matrix (*) and occasional in perimatrix (*), HβD–4 IHC, X 200. (**d**) Few HβD–4 positive cells in the epithelium (*) and none in the connective tissue (*) of a control skin sample, HβD–4 IHC, X 200 [38].

In the cholesteatoma group, we found anything from no (0) to moderate-to-numerous (++/+++) HβD-2- and HβD-4-positive cells. In the control group, the appearance and distribution of HβD-2- and HβD-4-containing cells ranged from none (0) to numerous (+++) (Figure 11a–d).

### 3.8. Statistical Analysis

Statistically significant differences in cell-positive factors between the patient and control groups are presented in Table 1.

The results of Spearman’s rank correlation between different factors of the cholesteatoma patient group are displayed in Table 2, demonstrating that there were statistically significant results.

## 4. Discussion

The main issue for patients with cholesteatoma is that it is a locally destructive lesion and causes degradation of the surrounding temporal bone, which can lead to several intra- or extra-temporal complications [18].

Therefore, the main issue associated with bone degradation is remodeling factors. Even though several authors have found overexpression of MMP-2 and MMP-9 in cholesteatoma tissue and linked these findings to the aggressiveness of the cholesteatoma [5,6,8], we, on the other hand, did not find any statistically significant difference between the expression of MMP-2 and MMP-9 in cholesteatoma compared to the control tissue. These findings are supported by Banerjee et al. and Rezende et al. [39,40], who also failed to find upregulation of these factors in cholesteatoma tissue. We suggest that more active functioning of TIMPs, in this case, might lead to remodulation of the tissue.

Although there were no statistically significant differences between TIMP-2 and TIMP-4 in the patient group compared to the control group, we found a slightly smaller number of TIMP-2-positive cells in the matrix than in the control skin epithelium, which was close to a statistically significant difference (*p* = 0.056). This might be a tendency, and the imbalance between MMPs and TIMPs could be the underlying mechanism of the aggressiveness of cholesteatoma. Similar findings are described by Schönermark et al., who mentioned an imbalance between MMPs and TIMPs cause proteolysis [9]. Furthermore, bone remodeling by MMP-2 and MPP-9 is strongly associated with angiogenesis [5,6,7,8]. Even though we did not find a statistically significant difference between VEGF in cholesteatoma and control skin, without any doubt, every researcher admits that angiogenesis in s cholesteatoma perimatrix is much more prominent than in skin connective tissue, as was proven by Olszewska et al. [41]. Additionally, angiogenesis supports the continuous growth of the cholesteatoma, which is also similarly found in different tumors [26,27]. We proved a strong positive correlation between MMP-2, MMP-9 and VEGF in the perimatrix, but such a correlation was absent in the control group. This finding might suggest that MMP-2 and MMP-9 intercorrelate with VEGF and cause pathological neo-angiogenesis in cholesteatoma tissue in children (still growing and developing tissue) [42]. Moreover, we found moderate and strong correlations between NF-κβ and VEGF in the patient group; as it is known that NF-κβ acts in a pathway to regulate the activity of VEGF in cholesteatoma [25,27], this might indicate complex intercorrelations between MMP-2, MMP-9, NF-κβ and VEGF, affecting angiogenesis in the cholesteatoma perimatrix.

Our study showed a statistically significant upregulation of the Shh gene protein in the cholesteatoma perimatrix. The Shh gene is a major factor contributing to correct craniofacial development in humans [43]. It also regulates the development of the external ear, from where epithelial cells migrate to the middle ear and form cholesteatoma [12,13,14,15,16]. The ongoing study’s authors suggest that the Shh gene might play a major role in the development of cholesteatoma. Thus, we suggest that the Shh gene might stimulate cholesteatoma growth in children.

To research inflammation processes in cholesteatoma, we detected pro- and anti-inflammatory cytokines IL-1 and IL-10. We did not find a statistically significant difference in the numbers of IL-1- and IL-10-positive cells between the patient and control groups, which is similar to the data of Yetiser et al. [44], who researched IL-1, and Kuczkowski et al. [45], who showed slight upregulation of IL-10 in cholesteatoma. Still, IL-10 in the cholesteatoma tissue did not statistically differ from the levels of IL-10 in the external ear canal skin. However, we found a strong positive correlation between IL-1 and IL-10 in cholesteatoma tissue and an opposite, very strong negative correlation between IL-1 and IL-10 in the control group. These findings might suggest dysregulation between IL-1 and IL-10 in cholesteatoma, and therefore, more favorable conditions for inflammatory processes in the cholesteatoma perimatrix, which may likely cause bone destruction. So, we believe that it is very important to measure both pro- and anti-inflammatory cytokines to examine the morphopathogenesis of cholesteatoma.

The most characteristic feature of cholesteatoma is its hyperproliferation of keratinocytes, which we can observe in the matrix and cystic layers of cholesteatoma [18]. To evaluate cell proliferation in our study, Ki-67 and NF-κβ were used. Even though many authors show that Ki-67 is upregulated in cholesteatoma [46,47], our results did not show statistically significant results between groups. Similar results with no statistically significant difference of Ki-67 in cholesteatoma compared to skin were presented by Kim et al. [48] and Kuczkowski et al. [49]. It is known that Ki-67 is found in all cell phases except G0 [23], and we believe that most of the keratinocytes in cholesteatoma are probably in the G0 phase and have either stopped the proliferation process or are ready to enter the G0/G1 transition to proliferate further. In addition, Chae et al. [50], in their study, proved that cell proliferation in cholesteatoma is controlled, and that the cell cycle can be stopped, compared to malignant tumors. Therefore, we suggest that Ki-67 in cholesteatoma is not the most reliable marker to show proliferation. This is also supported by Kim et al. [48], who found that other markers (not Ki-67) are more reliable to show cell proliferation in cholesteatoma. For instance, gankyrin, which is a p28 oncoprotein and is responsible for sustaining cell cycle progression [48]. However, Ki-67 is a good proliferation and prognostic marker in cancers, where the cells do not stop the proliferation process [51,52].

Our study showed that NF-κβ immunoreactive cells in the cholesteatoma matrix and perimatrix are noticeably found in greater numbers than in the control group, as proven by a statistically significant difference. These findings are supported by Byun et al. [53], who demonstrated upregulation on NF-κβ in cholesteatoma. NF-κβ and Ki-67 act through an inhibitor of the DNA binding protein 1 (Id1)→NF-κB→cyclin D1→Ki-67 signaling pathway to promote cell proliferation [25]. Our study also showed a strong positive correlation between NF-κβ and Ki-67, which proves that NF-κβ and Ki-67 are connected in the pathway to induce cell proliferation in cholesteatoma. Furthermore, NF-κβ helps to transit cholesteatoma cells from the G0 to the S phase [25]. The findings in our study allow us to conclude that NF-κβ might be a better cell marker to prove hyperproliferation in cholesteatoma than Ki-67. This suggestion is supported by Liu et al. [54] and Byun et al. [53].

Our study presented the upregulation of HβD-2 but found fewer HβD-4 immunoreactive cells in cholesteatoma compared to the control group, and these findings reached statistical significance. Similar results with upregulation of HβD-2 in their studies were shown by Park et al. [35] and Song et al. [55]. Acquired cholesteatoma is often accompanied by a chronic middle-ear infection. Most commonly, the inflammation is caused by *Pseudomonas aeruginosa* [32]. HβD-2 and HβD-4 have proven to form strong antibacterial defense mechanisms in the organism against these bacteria [33,34]. However, there are no studies on HβD-4 and cholesteatoma. Our study indicates that HβD-2 is the antibacterial peptide more expressed in cholesteatoma in comparison to HβD-4. It might be the first line of defense in cholesteatoma against bacterial superinfection [56]. Furthermore, it has been proven that IL-1 is a very effective inducer of HβD-2, and HβD-2 is increased in inflamed tissue [57,58]. Nonetheless, NF-κβ is essential for the induction of HβD-2 upon IL-1 stimulation [57]. In agreement with these findings, our study presents strong and very strong positive correlations between IL-1, NF-κβ and HβD-2, but not HβD-4.

Our study confirmed the significance of the complex research into different cell factors in cholesteatoma and revealed the most important of them: remodeling factors MMP-2, MMP9, TIMP-2 and TIMP-4; Shh gene protein; pro- and anti-inflammatory cytokines IL-1 and IL-10; cellular proliferation markers NF-κβ and Ki-67; angiogenetic factor VEGF and Human beta defensins 2 and 4. According to the revealed data, we can understand the level of complication of the pathogenesis of cholesteatoma. Similar studies using immunohistochemical evaluation of different tissue factors in retraction pockets of the tympanic membrane have been conducted, and it would be useful to compare and analyze the results between cholesteatoma and retraction pocket groups [59].

We realize that the present study has certain limitations. Additional quantification of tissue markers by standardized laboratory measurements (e.g., ELISA) would benefit the purely visual evaluation of immunohistochemically stained samples. Furthermore, we acknowledge that the relatively small control group and material taken from cadavers might pose limitations to the study. Additionally, partly our control group consist of adult cadaver skin material, which we compared to children cholesteatoma, might pose some limitations to the study. Ethical considerations, however, mandate the use of this relative control group. Moving forward, we encourage more studies to be undertaken to investigate different genes that might be responsible for the development of cholesteatoma in children.

## 5. Conclusions

The prominent TIMP, but not MMP, expression suggests probable suppression of tissue degradation in the cholesteatoma.

Complex intercorrelations between MMPs, NF-κβ and VEGF cause the intensification of angiogenesis in cholesteatoma perimatrix during childhood.

The persistent increase in Shh gene protein expression in the cholesteatoma perimatrix suggests the stimulation of tumor-affected tissue growth and development in children.

Similar expression of IL-1 and IL-10, and their strong positive intercorrelation, proves there is a balance between pro- and anti-inflammatory cytokines, despite the abundance of inflammatory cells.

NF-κβ, and not Ki-67, seems to be the main inducer of cellular proliferation in cholesteatoma.

The main antimicrobial protection is provided by HβD-2, upregulated by NF-κβ and IL-1, in cholesteatoma-affected tissue during childhood.

## Figures and Tables

**Table 1:** Mann–Whitney U test revealing statistically significant differences in positive cell factors between cholesteatoma patients and control group.

Detected Factor	Mann–Whitney U Test	Z-Score	*p*-Value
Shh perimatrix and Shh control connective tissue	25,500	−3180	0.001
NF-κβ matrix and NF-κβ control epithelium	21,500	−3332	0.001
NF-κβ perimatrix and NF-κβ control connective tissue	40,000	−2451	0.017
HβD-2 perimatrix and HβD-2 control connective tissue	22,500	−3326	0.001
HβD-4 matrix and HβD-4 control epithelium	167,500	4001	0.000

Abbreviations: Shh—Sonic hedgehog; NF-κβ—nuclear factor kappa beta; HβD-2—human beta defensin 2; HβD-4—human beta defensin 4.

**Table 2:** Spearman’s rank correlation coefficient revealed correlations between the relative numbers of different tissue factors in the cholesteatoma matrix and perimatrix.

Markers		MMP-2 M	MMP-2 P	MMP-9 M	MMP-9 P	TIMP-2 M	TIMP-2 P	TIMP-4 M	TIMP-4 P	Shh M	Shh P	IL-1 M	IL-1 P	IL-10 M	IL-10 P	NF-κβ M	NF-κβ P	Ki-67 M	Ki-67 P	VEGF M	VEGF P	HβD2 M	HβD2 P	HβD4 M	HβD4 P
MMP-2 M	Rs p																								
MMP-2 P	Rs p	0.824 ** 0.000																							
MMP-9 M	Rs p	0.265 0.320	0.487 0.056																						
MMP-9 P	Rs p	0.212 0.430	0.471 0.066	0.701 ** 0.002																					
TIMP-2 M	Rs p	0.710 ** 0.002	0.615 * 0.011	0.465 0.070	0.363 0.167																				
TIMP-2 P	Rs p	0.612 * 0.012	0.793 ** 0.000	0.587 * 0.017	0.678 ** 0.004	0.718 ** 0.002																			
TIMP-4 M	Rs p	0.366 0.163	0.513 * 0.042	0.547 * 0.028	0.371 0.157	0.145 0.592	0.370 0.158																		
TIMP-4 P	Rs p	0.197 0.466	0.354 0.179	0.286 0.282	0.198 0.463	−0.099 0.715	0.134 0.620	0.747 ** 0.001																	
Shh M	Rs p	0.337 0.202	0.435 0.093	0.493 0.053	0.447 0.083	0.248 0.354	0.440 0.088	0.745 ** 0.001	0.463 0.071																
Shh P	Rs p	0.462 0.072	0.535 * 0.033	0.494 0.052	0.383 0.143	0.488 0.055	0.610 * 0.012	0.538 * 0.032	0.366 0.164	0.845 ** 0.000															
IL-1 M	Rs p	0.327 0.216	0.288 0.280	0.195 0.468	0.170 0.530	0.187 0.488	0.199 0.460	0.702 ** 0.002	0.594 * 0.015	0.576 * 0.020	0.286 0.284														
IL-1 P	Rs p	−0.040 0.883	0.237 0.376	0.597 * 0.015	0.646 ** 0.007	0.176 0.514	0.454 0.077	0.493 0.052	0.487 0.056	0.323 0.222	0.230 0.391	0.318 0.229													
IL-10 M	Rs p	0.308 0.246	0.302 0.255	0.006 0.982	0.047 0.864	0.320 0.226	0.290 0.276	0.269 0.313	0.368 0.161	0.599 * 0.014	0.580 * 0.019	0.543 * 0.030	0.177 0.513												
IL-10 P	Rs p	0.118 0.663	0.252 0.346	0.255 0.341	0.248 0.354	0.074 0.784	0.258 0.336	0.782 ** 0.000	0.811 ** 0.000	0.588 * 0.016	0.446 0.083	0.707 ** 0.002	0.594 * 0.015	0.466 0.069											
NF-κβ M	Rs p	0.312 0.239	0.387 0.139	0.334 0.206	0.382 0.144	0.133 0.624	0.189 0.483	0.700 ** 0.003	0.663 ** 0.005	0.734 ** 0.001	0.486 0.056	0.768 ** 0.001	0.246 0.358	0.526 * 0.036	0.640 ** 0.008										
NF-κβ P	Rs p	−0.167 0.535	0.112 0.680	0.328 0.215	0.442 0.087	0.143 0.598	0.350 0.183	0.258 0.334	0.254 0.343	0.509 * 0.044	0.502 * 0.048	0.291 0.274	0.370 0.159	0.403 0.122	0.432 0.095	0.552 * 0.027									
Ki-67 M	Rs p	0.112 0.679	0.118 0.664	0.243 0.364	0.043 0.873	0.141 0.603	0.184 0.495	0.434 0.093	0.455 0.077	0.706 ** 0.002	0.712 ** 0.002	0.507 * 0.045	0.246 0.359	0.641 ** 0.007	0.533 * 0.033	0.566 * 0.022	0.631 ** 0.009								
Ki-67 P	Rs p	−0.095 0.727	0.028 0.919	0.095 0.727	0.230 0.390	−0.144 0.595	0.150 0.580	0.404 0.121	0.253 0.345	0.671 ** 0.004	0.487 0.056	0.333 0.208	0.250 0.350	0.376 0.152	0.439 0.089	0.546 * 0.029	0.720 ** 0.002	0.687 ** 0.003							
VEGF M	Rs p	0.019 0.944	0.344 0.193	0.562 * 0.023	0.657 ** 0.006	0.235 0.380	0.435 0.092	0.445 0.084	0.380 0.146	0.528 * 0.036	0.487 0.056	0.225 0.402	0.398 0.127	0.230 0.392	0.497 0.050	0.623 ** 0.010	0.760 ** 0.001	0.273 0.305	0.424 0.101						
VEGF P	Rs p	0.296 0.266	0.618 * 0.011	0.684 ** 0.004	0.713 ** 0.002	0.305 0.250	0.632 ** 0.009	0.764 ** 0.001	0.470 0.066	0.573 * 0.020	0.494 0.052	0.407 0.118	0.563 * 0.023	0.053 0.845	0.508 * 0.045	0.571 * 0.021	0.526 * 0.036	0.234 0.384	0.403 0.122	0.675 ** 0.004					
HβD-2 M	Rs p	−0.048 0.860	0.045 0.868	0.202 0.453	0.262 0.327	0.037 0.890	0.085 0.754	0.536 * 0.032	0.437 0.091	0.675 ** 0.004	0.375 0.152	0.671 ** 0.004	0.364 0.166	0.653 ** 0.006	0.604 * 0.013	0.738 ** 0.001	0.578 * 0.019	0.501 * 0.048	0.616 * 0.011	0.516 * 0.041	0.386 0.140				
HβD-2 P	Rs p	0.351 0.182	0.390 0.135	0.171 0.525	0.216 0.421	0.384 0.142	0.511 * 0.043	0.448 0.081	0.261 0.329	0.680 ** 0.004	0.560 * 0.024	0.664 ** 0.005	0.350 0.184	0.847 ** 0.000	0.499 * 0.049	0.436 0.091	0.347 0.188	0.552 * 0.027	0.419 0.106	0.165 0.540	0.265 0.321	0.649 ** 0.007			
HβD-4 M	Rs p	0.426 0.100	0.373 0.155	0.142 0.601	0.301 0.258	0.441 0.088	0.591 * 0.016	0.052 0.847	0.200 0.457	0.356 0.176	0.650 ** 0.006	0.006 0.983	0.233 0.386	0.473 0.064	0.219 0.415	0.148 0.585	0.401 0.124	0.489 0.055	0.387 0.138	0.228 0.396	0.155 0.565	0.118 0.662	0.366 0.163		
HβD-4 P	Rs p	0.426 0.100	0.373 0.155	0.142 0.601	0.301 0.258	0.441 0.088	0.591 * 0.016	0.052 0.847	0.200 0.457	0.356 0.176	0.650 ** 0.006	0.006 0.983	0.233 0.386	0.473 0.064	0.219 0.415	0.148 0.585	0.401 0.124	0.489 0.055	0.387 0.138	0.228 0.396	0.155 0.565	0.118 0.662	0.366 0.163	0.426 0.100	

Abbreviations: Rs—Spearman’s correlation coefficient; *p*—*p*-value; M—Matrix; P—Perimatrix; MMP-2– matrix metalloproteinase 2; MMP-9—matrix metalloproteinase 9; TIMP-2—tissue inhibitor of metalloproteinase-2; TIMP-4—tissue inhibitor of metalloproteinase-4; Shh—Sonic hedgehog gene protein; IL-1—Interleukin 1; IL-10—Interleukin 10; NF-κβ—nuclear factor kappa beta; Ki-67—proliferation marker; VEGF—vascular endothelial growth factor; HβD-2—Human beta defensin 2; HβD-4—Human beta defensin 4; * Correlation is significant at the 0.05 level (2-tailed); ** Correlation is significant at the 0.01 level (2-tailed).

## Data Availability

The datasets used and/or analyzed during the current study are presented in the results section of the present study.
